# Supplementary material for: SARS-CoV-2 S Mutations: A Lesson from the Viral World to Understand How Human Furin Works
Source: Int J Mol Sci. 2023 Mar 1;24(5):4791. doi: 10.3390/ijms24054791 (PMC10003014; doi:10.3390/ijms24054791)
Supplement: Supplementary file 1 [file ijms-24-04791-s001.zip › Supplementary materials.pdf]

SUPPLEMENTARY FIGURES

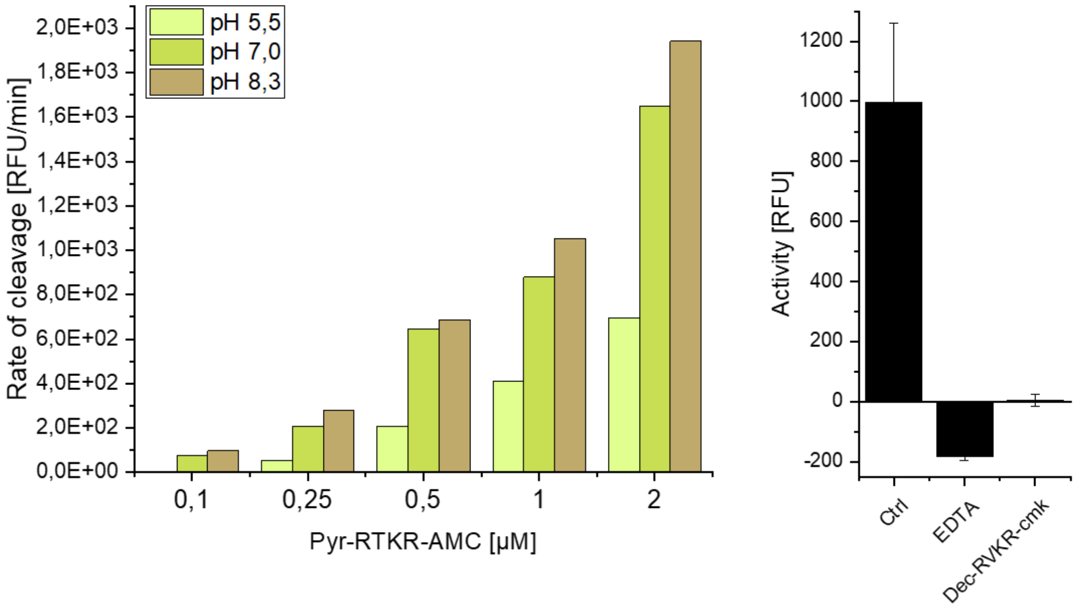

Fig. S1 Human soluble Furin activity and specificity. Recombinant soluble hFur was incubated with increasing concentrations of the fluorogenic Pyroglutamic (Pyr)-RTKR- Methyl amino coumarin (AMC) peptide at diggerent pHs. Relative fluorescence units (RFU) were measured over time (λ_{ex}=360nm; λ_{em}=460nm). Rate of substrate cleavage (RFU/min) was plotted over substrate concentration (left). Data confirm that hFur showes higher activity at neutral/basic pHs. Upon incubation with the generic Ethylenediaminetetraacetic acid (EDTA) inhibitor (10mM) or the Furin specific Decanoyl (Dec)-RVKR-chloro methyl ketone (cmk) inhibitor (2µM), soluble hFurin activity is fully inhibited.

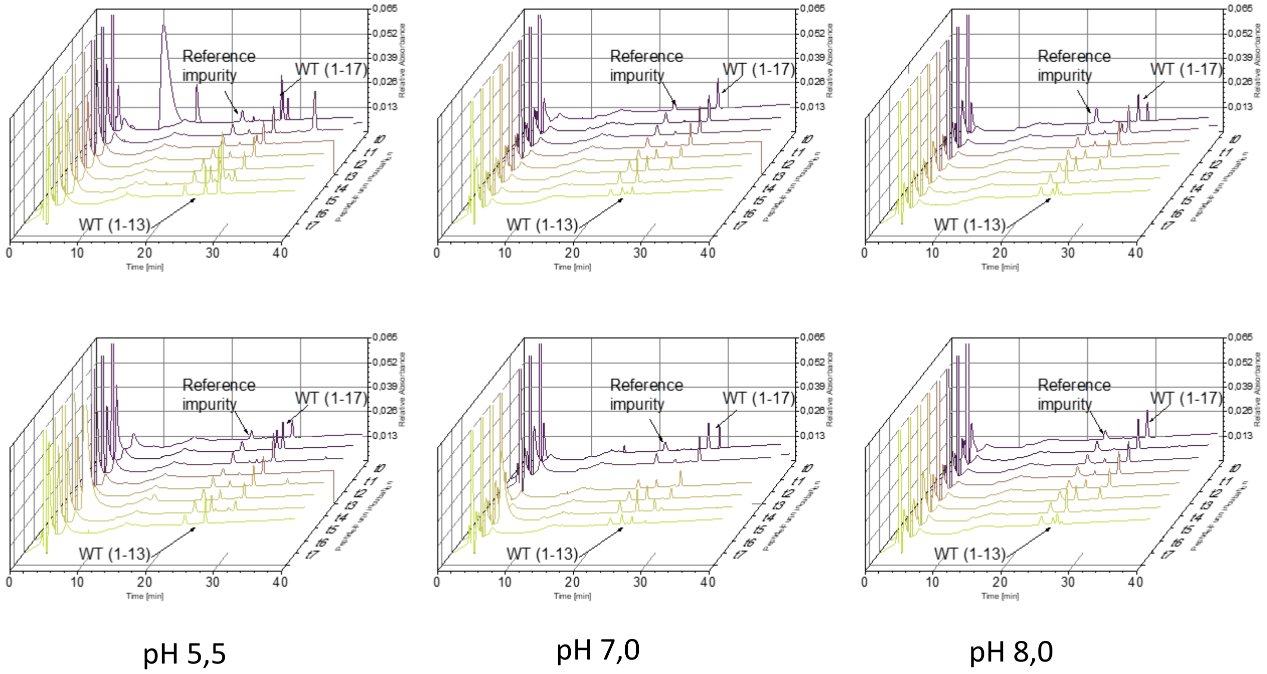

Fig. S2 Chromatograms of WT SARS-CoV-2 peptide digestion with sFur at pH 5,5, pH 7,0, and pH 8,0. WT SARS-CoV-2 peptide was incubated with recombinant sFur in duplicates (top and bottom) and samples collected at different time points and analyzed by HPLC (detection UV, 214 nm). Full length WT peptide (1-17), the generated fragment (1-13) following cleavage at the RRAR↓ motif and an impurity reference peak are indicated.

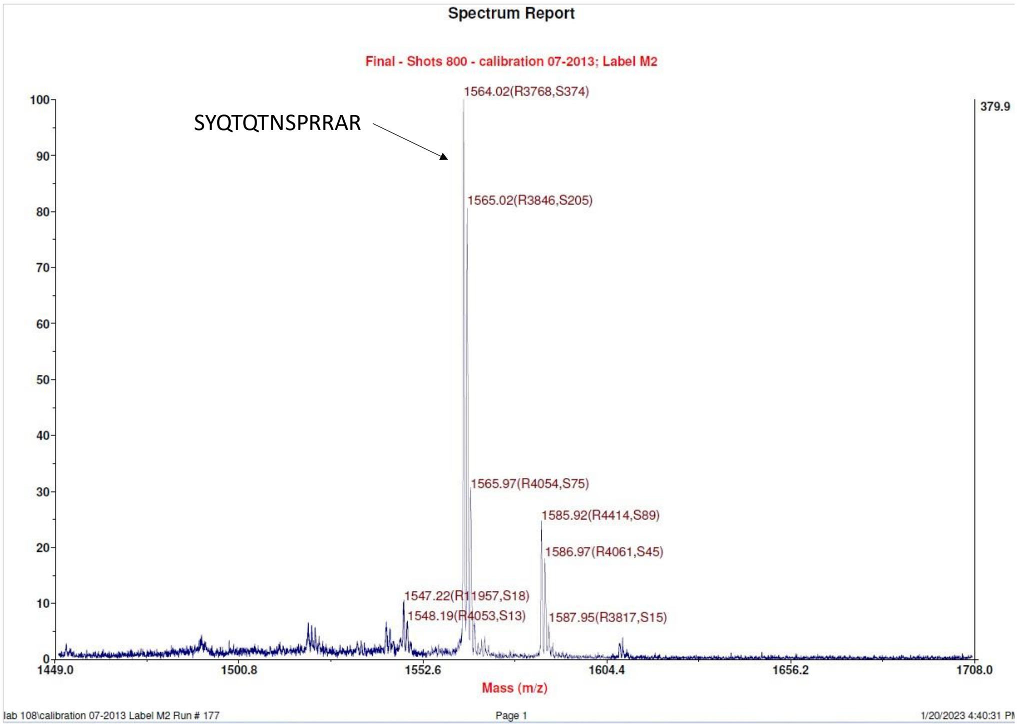

Fig. S3 Mass spectrometry analysis of WT SARS-CoV-2 peptide digestion. Peptide fragment (SYQTQTNSPRRAR, Experimental mass: 1564.09 Da, Theoretical mass: 1564.67 Da) relative to the sFur digestion is indicated following matrix-assisted laser desorption/ionization (MALDI) analysis (4800 MALDI-TOF/TOF TM analyzer provided with 4000 Series Explorer TM software, Applied Biosystem/MDS Sciex, Foster City, CA, USA).

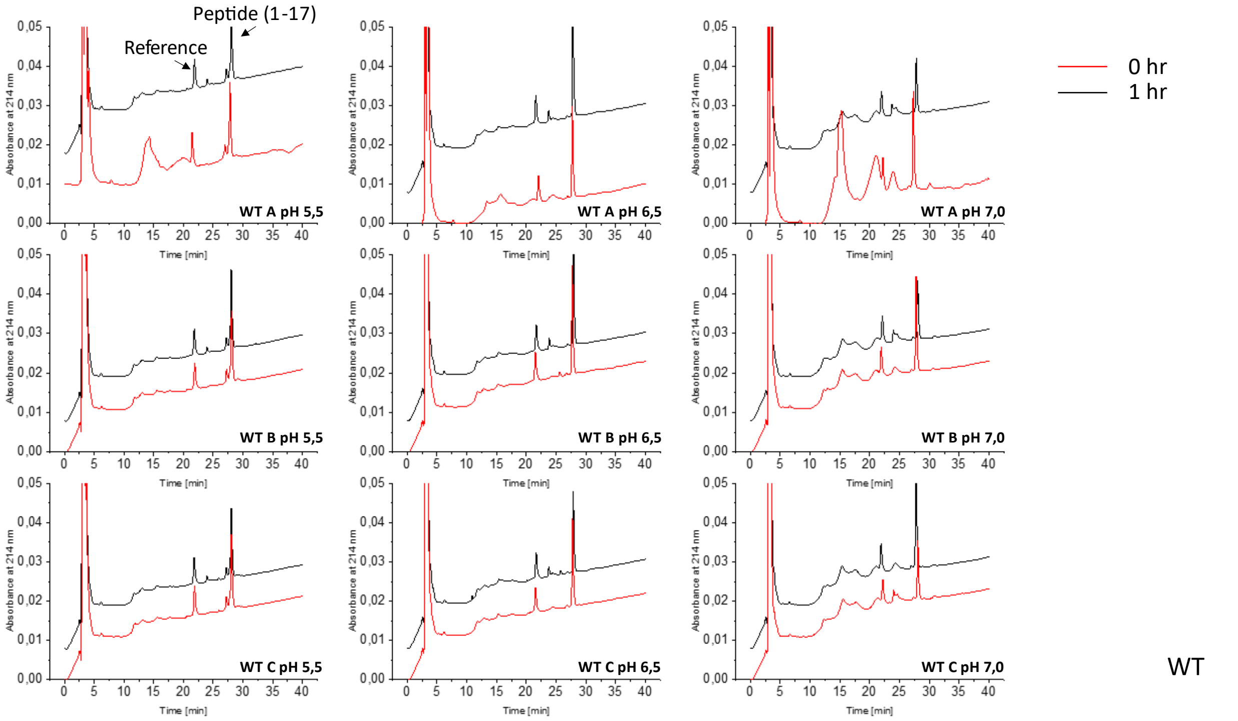
Peptide (1-17)
Reference
0 hr
1 hr
Absorbance at 214 nm
Time [min]
WT A pH 5,5
WT A pH 6,5
WT A pH 7,0
WT B pH 5,5
WT B pH 6,5
WT B pH 7,0
WT C pH 5,5
WT C pH 6,5
WT C pH 7,0
WT

(a)

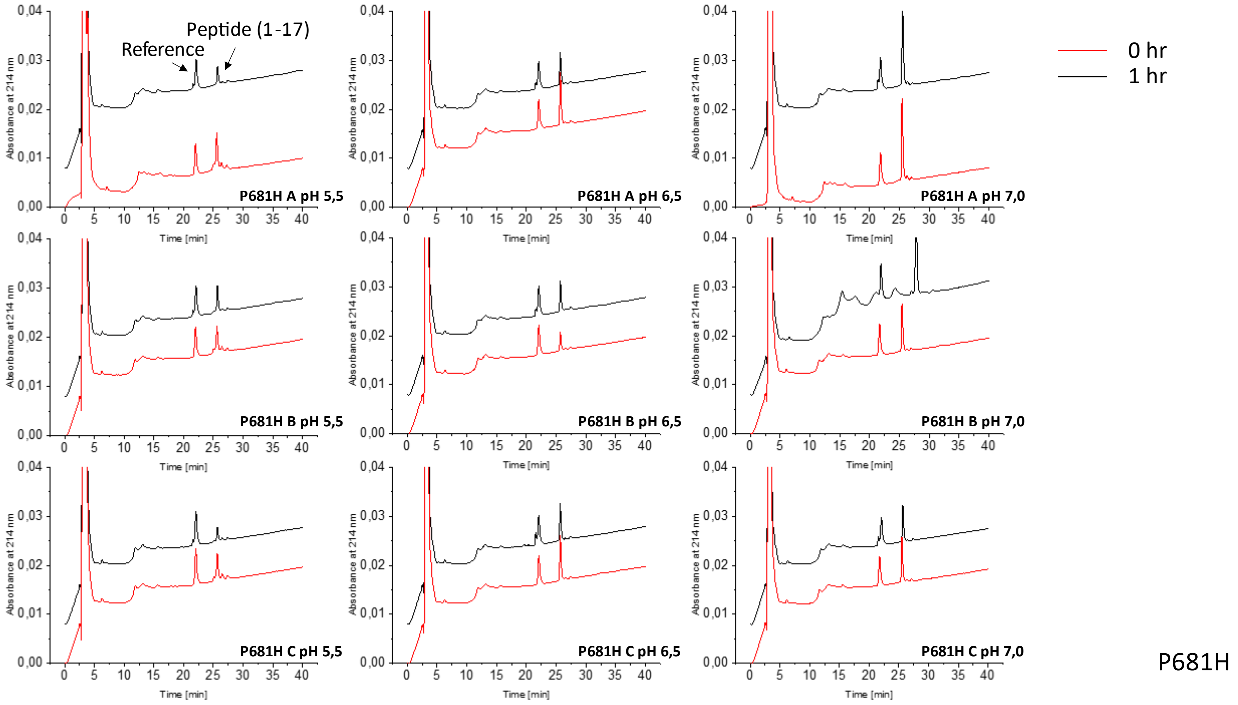
Peptide (1-17)
Reference
0 hr
1 hr
Absorbance at 214 nm
Time [min]
P681H A pH 5,5
P681H A pH 6,5
P681H A pH 7,0
P681H B pH 5,5
P681H B pH 6,5
P681H B pH 7,0
P681H C pH 5,5
P681H C pH 6,5
P681H C pH 7,0
P681H

(b)

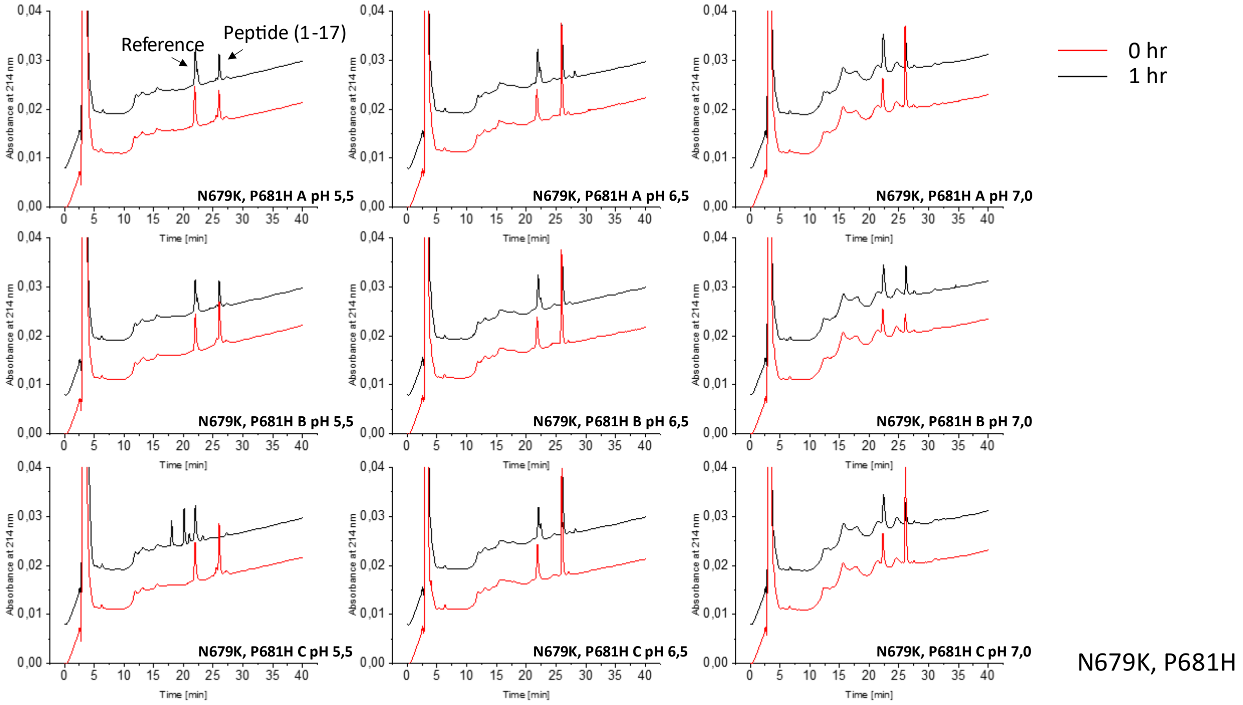
Reference
Peptide (1-17)
0 hr
1 hr
N679K, P681H A pH 5,5
N679K, P681H A pH 6,5
N679K, P681H A pH 7,0
N679K, P681H B pH 5,5
N679K, P681H B pH 6,5
N679K, P681H B pH 7,0
N679K, P681H C pH 5,5
N679K, P681H C pH 6,5
N679K, P681H C pH 7,0
Absobance at 214 nm
Time [min]
N679K, P681H

(c)

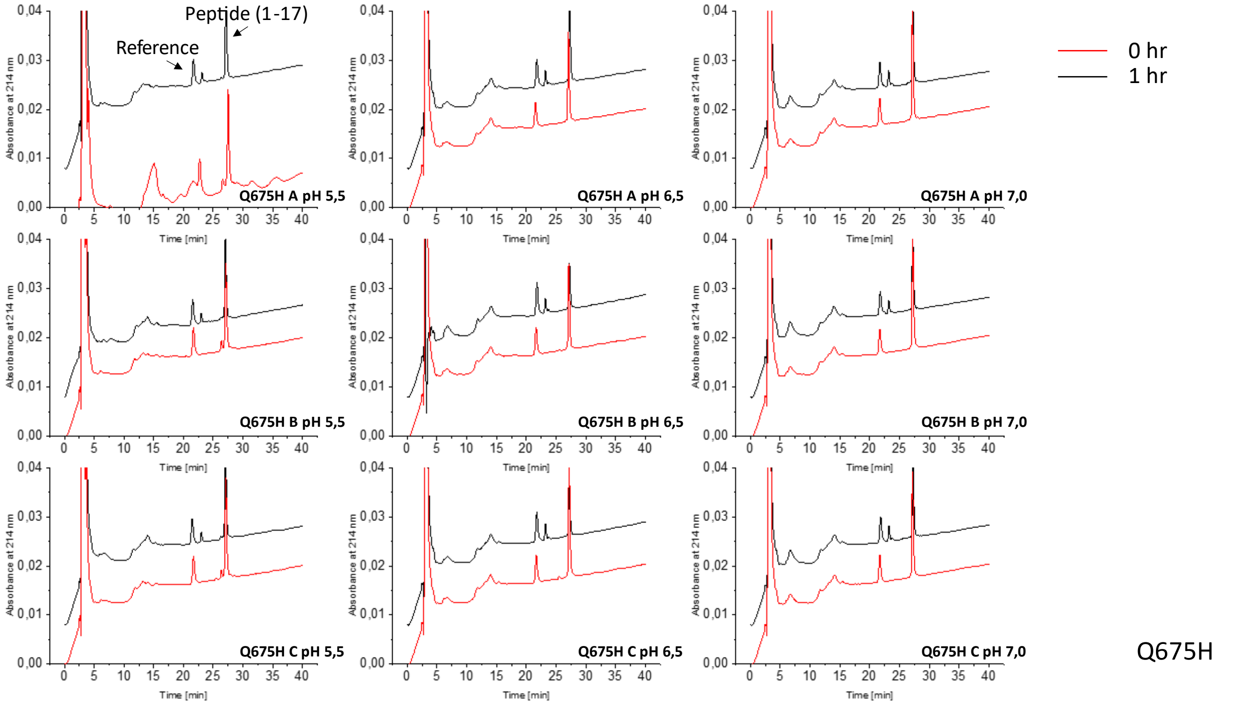
Peptide (1-17)
Reference
0 hr
1 hr
Q675H A pH 5,5
Q675H A pH 6,5
Q675H A pH 7,0
Q675H B pH 5,5
Q675H B pH 6,5
Q675H B pH 7,0
Q675H C pH 5,5
Q675H C pH 6,5
Q675H C pH 7,0
Absobance at 214 nm
Time [min]
Q675H

(d)

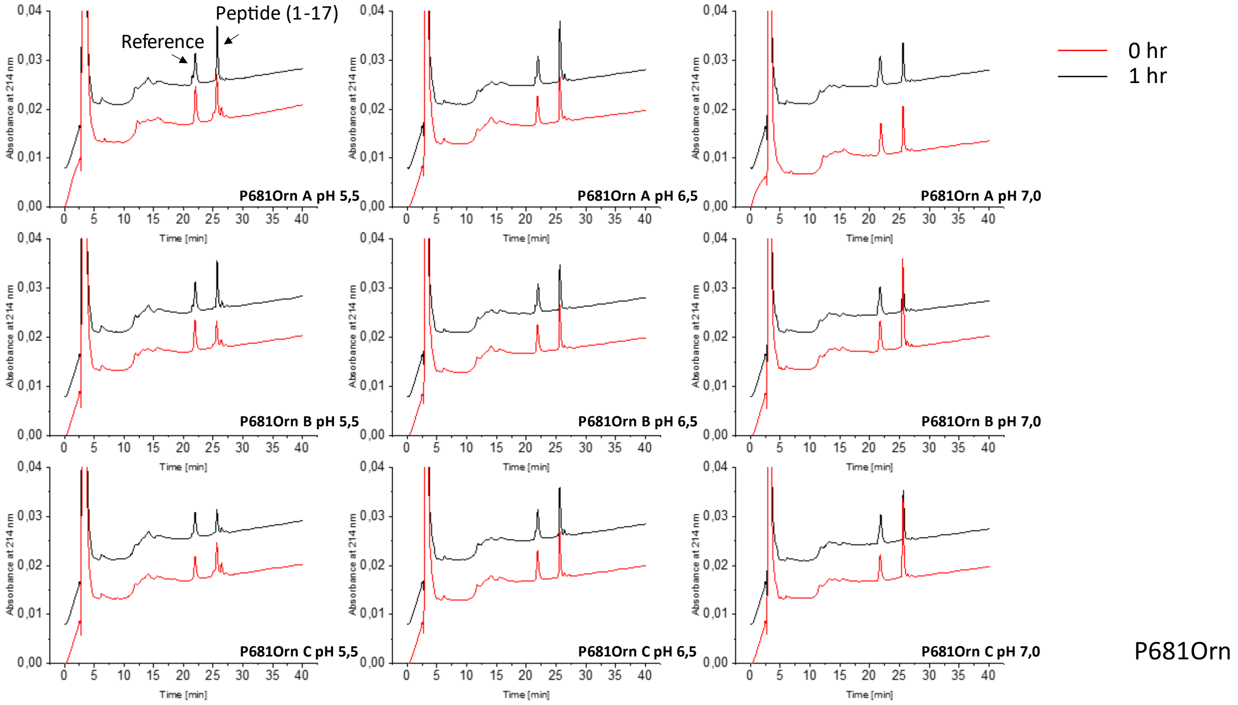
Peptide (1-17)
Reference
0 hr
1 hr
P681Orn A pH 5,5
P681Orn A pH 6,5
P681Orn A pH 7,0
P681Orn B pH 5,5
P681Orn B pH 6,5
P681Orn B pH 7,0
P681Orn C pH 5,5
P681Orn C pH 6,5
P681Orn C pH 7,0
P681Orn
Absorbance at 214 nm
Time [min]

(e)

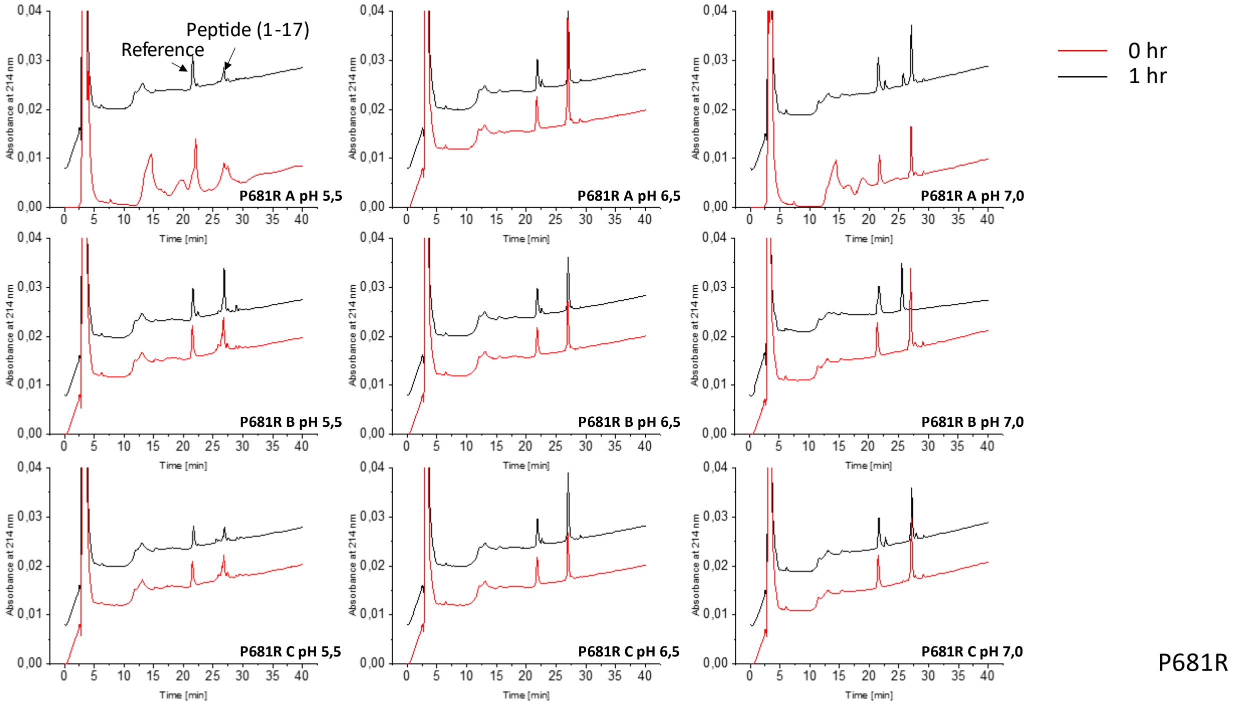
Peptide (1-17)
Reference
0 hr
1 hr
P681R A pH 5,5
P681R A pH 6,5
P681R A pH 7,0
P681R B pH 5,5
P681R B pH 6,5
P681R B pH 7,0
P681R C pH 5,5
P681R C pH 6,5
P681R C pH 7,0
P681R
Absorbance at 214 nm
Time [min]

(f)

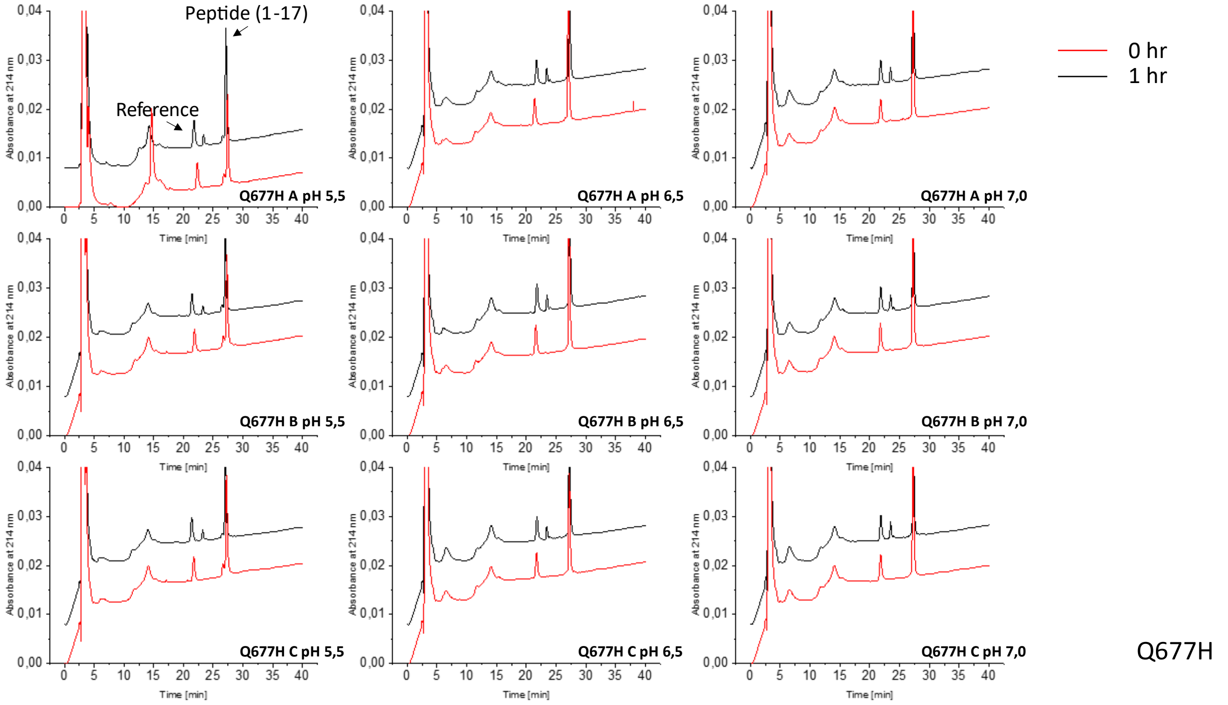
Peptide (1-17)
Reference
0 hr
1 hr
Q677H A pH 5,5
Q677H A pH 6,5
Q677H A pH 7,0
Q677H B pH 5,5
Q677H B pH 6,5
Q677H B pH 7,0
Q677H C pH 5,5
Q677H C pH 6,5
Q677H C pH 7,0
Q677H
Absorbance at 214 nm
Time [min]

(g)

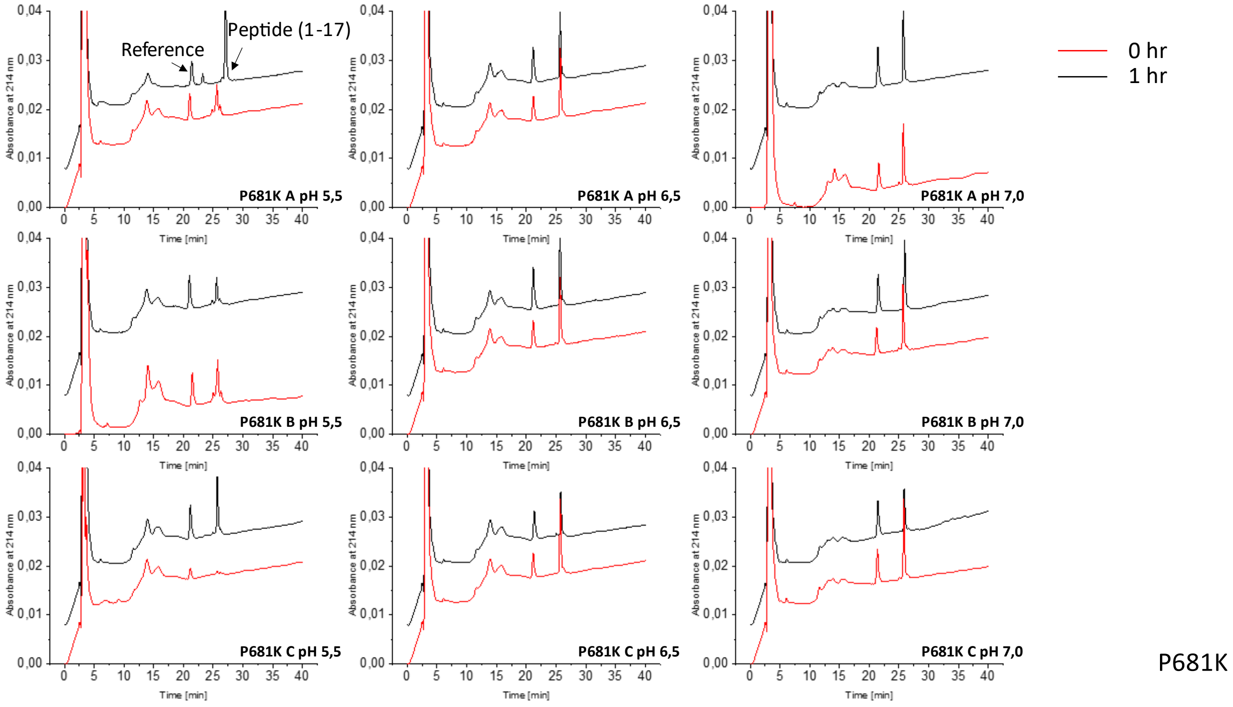
Reference
Peptide (1-17)
0 hr
1 hr
P681K A pH 5,5
P681K A pH 6,5
P681K A pH 7,0
P681K B pH 5,5
P681K B pH 6,5
P681K B pH 7,0
P681K C pH 5,5
P681K C pH 6,5
P681K C pH 7,0
P681K
Absorbance at 214 nm
Time [min]

(h)

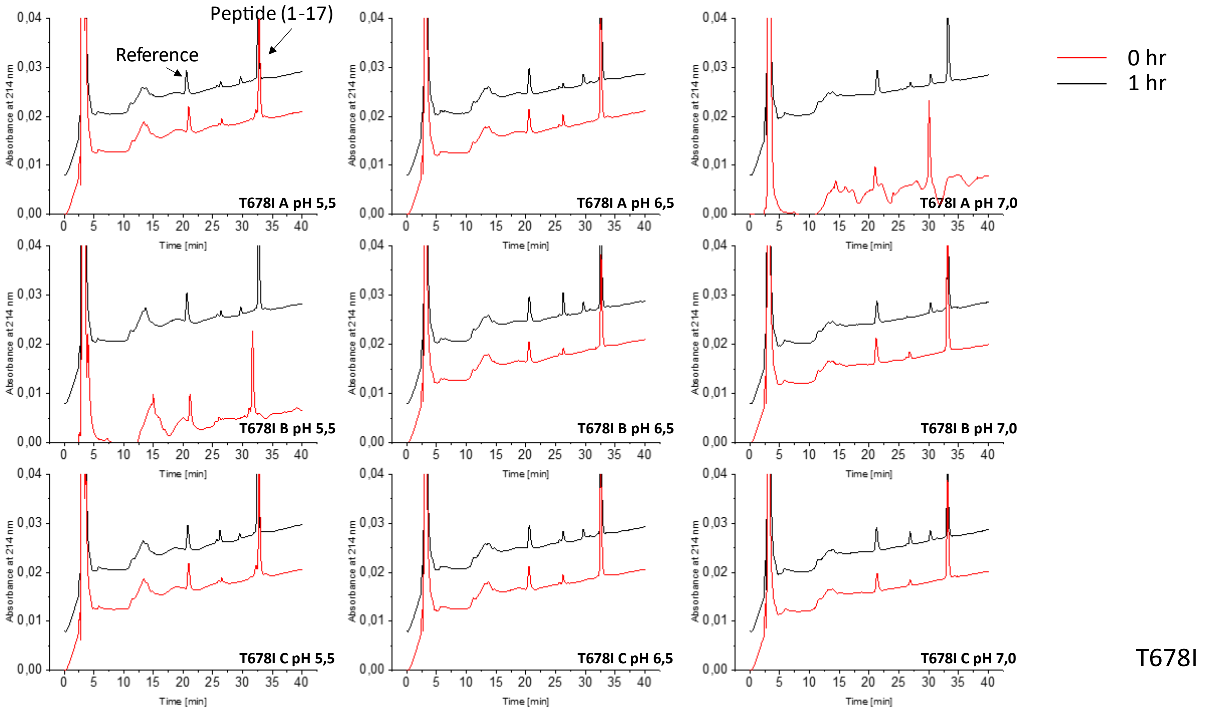

(i)

Fig. S4 : Chromatograms of peptides described in Tab.1 digested with sFur. SARS-CoV-2 peptides (WT (a), P681H (b), N679K, P681H (c), Q675H (d), P681Orn (e), P681R (f), Q677H (g), P681K (h), and T678I) were incubated with recombinant sFur (triplicate experiments, top, middle and bottom) and samples collected at time 0 (red) and 1hr (black) for following HPLC analysis (detection UV, 214 nm). Full length peptides (1-17) and impurity reference peak are indicated.
